# Intracranial hemorrhage in a patient with Urbach-Wiethe disease

**DOI:** 10.1055/s-0044-1789227

**Published:** 2024-09-02

**Authors:** Ana Luisa de Carvalho Cardozo Hernández, Gabriel Saboia de Araújo Torres, Thiago Trajano da Silva, Gisela Tinone, Lucas Fernandes Ferreira, João Paulo Motta Telles, Fernando Freua, Leandro Tavares Lucato

**Affiliations:** 1 Universidade de São Paulo, Faculdade de Medicina, Departamento de Neurologia, São Paulo SP, Brazil.; 2 Universidade de São Paulo, Faculdade de Medicina, Departamento de Neurorradiologia, São Paulo SP, Brazil.

**Figure 1:**
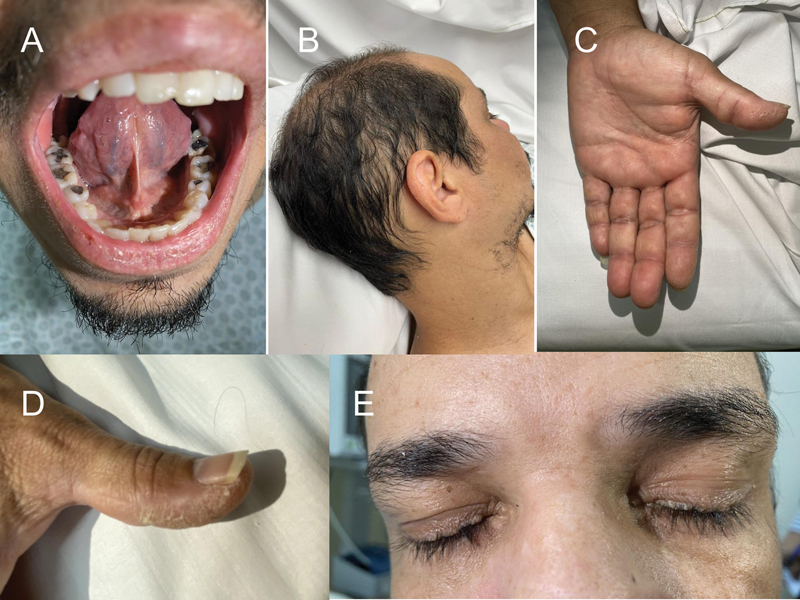
Signs related to Urbach-Wiethe disease: ( **A** ) Dry skin and hyperkeratotic plaques on the left hand. ( **B** ) Thickened tongue frenulum. ( **C** ) Early alopecia on the top of the scalp and decreased hair density. ( **D** ) Beaded papular lesions on the eyelids, resembling a pearl necklace, also known as moniliform blepharosis.

**Figure 2:**
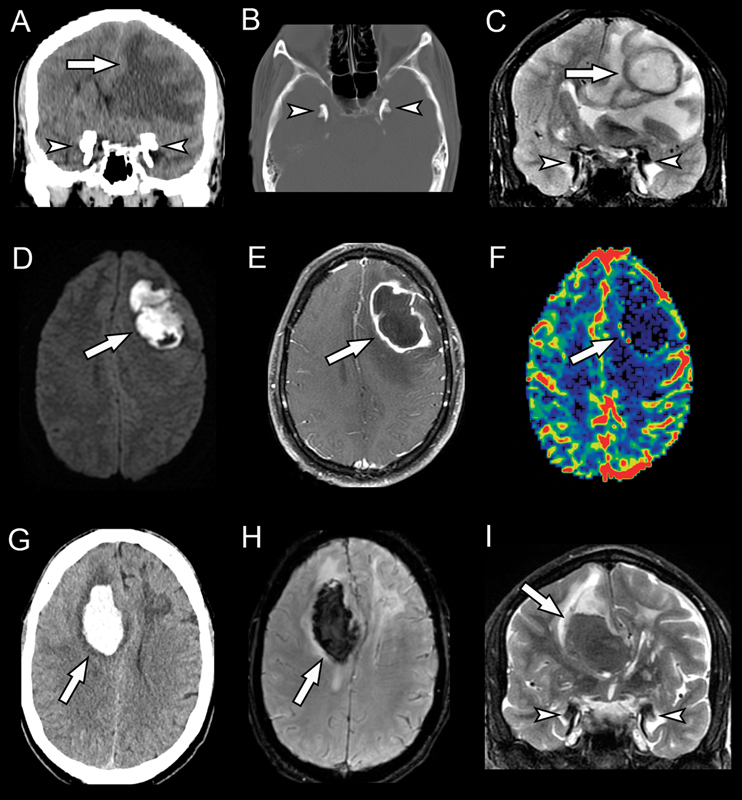
A noncontrast CT scan ( **A** ) demonstrates amygdalae comma-shaped calcifications (arrowheads) and hypoattenuation in left frontal lobe (arrow). A CT scan of the bone window ( **B** ) also shows calcifications (arrowheads). The coronal T2WI ( **C** ) depicts hypointense calcifications (arrowheads) and a lesion in left frontal lobe (arrow). Both DWI ( **D** ) and postcontrast T1WI ( **E** ) show central restricted diffusion and peripheral enhancement; no significant change in CBV color maps ( **F** ) is noted (arrows), suggesting an abscess. After 4 years the patient returned with an intraparenchymal hemorrhage in the right frontal lobe, seen in the noncontrast CT scan ( **G** ), SWI ( **H** ), and coronal T2WI ( **I** ) (arrows). Again, there are signs of calcification (arrowheads in **I** ).

A 39-year-old man presented with headache, left hemiparesis, and apathy. He also had epilepsy and hoarseness since infancy, tongue frenulum thickening, and yellow papular lesions around his eyelids and fingers ( Figure 1 ). The computed tomography (CT) scan showed right frontal lobe hemorrhage, with “comma” shaped calcifications in both amygdalae, a characteristic finding of the Urbach-Wiethe disease ( Figure 2 ). Previously, he presented with an intracranial pyogenic abscess due to poor dentition, perhaps related to hyaline deposits found along the parotid duct in these patients. 1 The whole exome sequencing shows a biallelic pathogenic variant c.816_817del (p.Cys272*) in the *ECM1* ( Figure 2 ). Urbach-Wiethe disease is an autosomal recessive disorder, with hyaline-like material deposition in the skin, mucosae, and viscera. 2 3 Neurological features include seizures, neuropsychiatric manifestations, intracranial hemorrhages, and calcifications in the mesial temporal lobes. 4
